# The role of lipid second messengers in aldosterone synthesis and secretion

**DOI:** 10.1016/j.jlr.2022.100191

**Published:** 2022-03-10

**Authors:** Shinjini C. Spaulding, Wendy B. Bollag

**Affiliations:** 1 Department of Physiology, Medical College of Georgia at Augusta University, Augusta, GA, USA; 2 Research Department, Charlie Norwood VA Medical Center, Augusta, GA, USA

**Keywords:** adrenal cortex, glycerophospholipids, intracellular signaling, signal transduction, sphingolipids, steroidogenesis, phospholipases, primary aldosteronism, 3βHSD2, type II 3β-hydroxysteroid dehydrogenase, AA, arachidonic acid, ACSL4, acyl-CoA synthetase long chain family member 4, Ang II, angiotensin II, C1P, ceramide-1-phosphate, CYP11A1, cholesterol side-chain cleavage complex, CYP21, 21-Hydroxylase, DAG, diacylglycerol, DGKθ, DAG kinase θ, IP_3_, inositol 1,4,5-trisphosphate, LPA, lysophosphatidic acid, LPP, lipid phosphate phosphatase, PA, phosphatidic acid, PLA_2_, phospholipase A_2_, PLC, phospholipase C, PEt, phosphatidylethanol, PIP_2_, phosphatidylinositol 4,5-bisphosphate, PKC, protein kinase C, PKD, protein kinase D, PP1, protein phosphatase 1, PTH, parathyroid hormone, PTP, protein tyrosine phosphatase, PTX, pertussis toxin, RSK, ribosomal S6 kinase, S1P, sphingosine-1-phosphate, SF-1, steroidogenic factor-1, SGPL1, S1P lyase 1, SPC, sphingosylphosphorylcholine, TASK, TWIK-related-acid-sensitive potassium, zG, zona glomerulosa

## Abstract

Second messengers are small rapidly diffusing molecules or ions that relay signals between receptors and effector proteins to produce a physiological effect. Lipid messengers constitute one of the four major classes of second messengers. The hydrolysis of two main classes of lipids, glycerophospholipids and sphingolipids, generate parallel profiles of lipid second messengers: phosphatidic acid (PA), diacylglycerol (DAG), and lysophosphatidic acid versus ceramide, ceramide-1-phosphate, sphingosine, and sphingosine-1-phosphate, respectively. In this review, we examine the mechanisms by which these lipid second messengers modulate aldosterone production at multiple levels. Aldosterone is a mineralocorticoid hormone responsible for maintaining fluid volume, electrolyte balance, and blood pressure homeostasis. Primary aldosteronism is a frequent endocrine cause of secondary hypertension. A thorough understanding of the signaling events regulating aldosterone biosynthesis may lead to the identification of novel therapeutic targets. The cumulative evidence in this literature emphasizes the critical roles of PA, DAG, and sphingolipid metabolites in aldosterone synthesis and secretion. However, it also highlights the gaps in our knowledge, such as the preference for phospholipase D-generated PA or DAG, as well as the need for further investigation to elucidate the precise mechanisms by which these lipid second messengers regulate optimal aldosterone production.

Extracellular signals, received and transduced by receptors at the cell surface, elicit cellular responses via the generation of small, rapidly diffusing molecules referred to as second messengers. Second messengers are intermediates that link extracellular signals to intracellular responses. Lipid second messengers, produced by the metabolism of lipids, are one of the major classes of second messengers. The purpose of this review is to highlight the role of lipid second messengers in regulating aldosterone production in the adrenal cortex.

## Aldosterone biosynthesis

Aldosterone is the principal mineralocorticoid hormone synthesized in and secreted from the zona glomerulosa (zG) layer of the adrenal cortex, primarily in response to angiotensin II (Ang II), elevated serum potassium levels, and adrenocorticotrophic hormone (ACTH). It plays a central role in electrolyte and fluid volume regulation and maintenance of blood pressure homeostasis and is tightly regulated by the renin-angiotensin-aldosterone system. Primary aldosteronism, in which plasma aldosterone levels are normal or elevated relative to suppressed plasma renin levels, is the most frequent cause of secondary hypertension. Primary aldosteronism accounts for 5%–10% of hypertension cases and up to 20% in the case of resistant hypertension (1), with the reported prevalence showing high variability among studies depending upon the population of patients included, the diagnostic criteria, and the severity of hypertension (2, 3, 4). Aldosterone has also been suggested to be one of the causal links between obesity and hypertension (5, 6, 7).

**Fig. 1:**
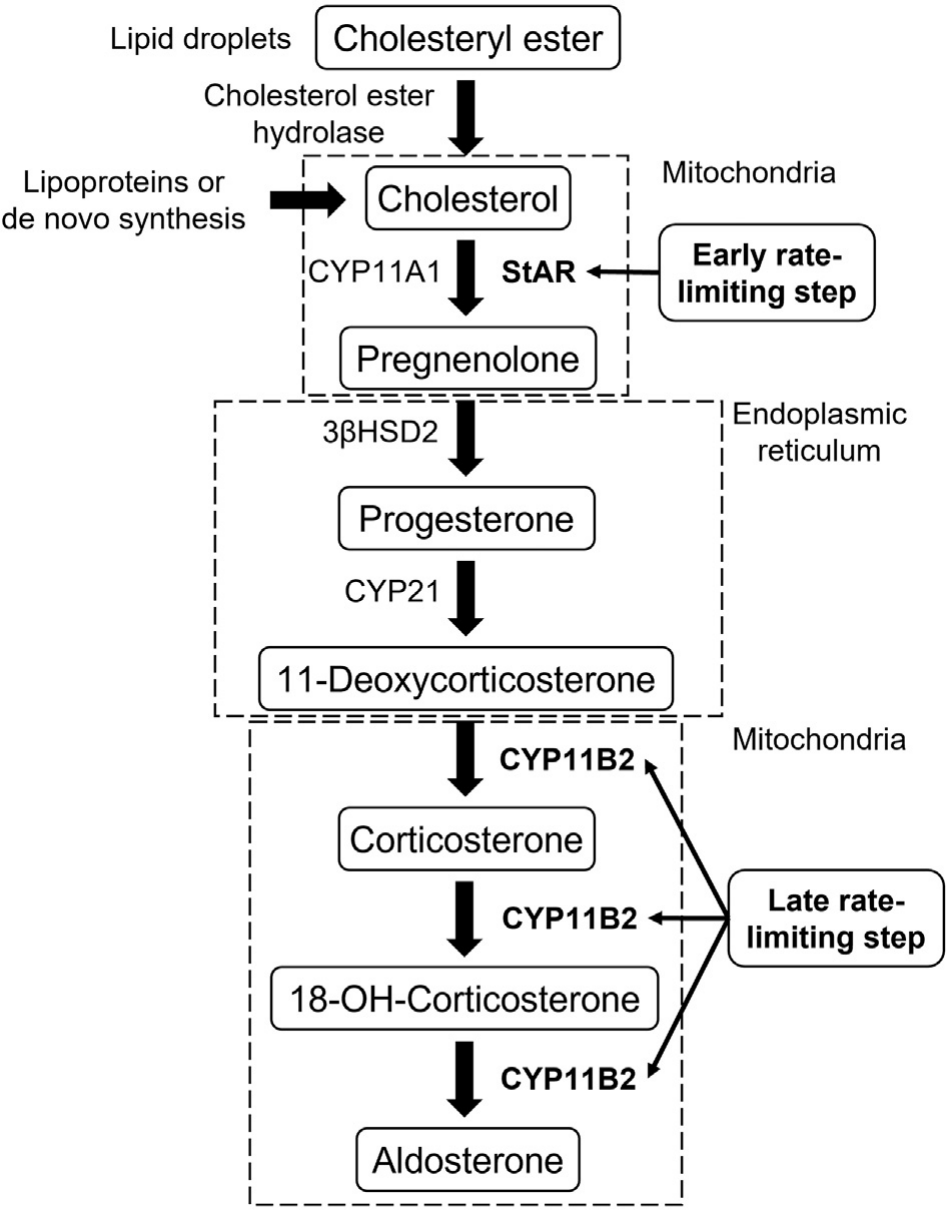
Aldosterone biosynthesis. This schematic illustrates the enzymatic process through which aldosterone is synthesized in the mitochondria and endoplasmic reticulum of zona glomerulosa cells in the adrenal cortex. The cholesterol precursor can be derived from a combination of sources: mobilization of cholesteryl esters stored in lipid droplets by cholesteryl ester hydrolase, de novo synthesis in the endoplasmic reticulum, and receptor-mediated uptake and internalization of plasma lipoprotein-derived cholesterol. The free cholesterol is transported by the steroidogenic acute regulatory (StAR) protein from the outer to the inner mitochondrial membrane, which is the early rate-limiting step in steroidogenesis. In the inner mitochondrial membrane, steroidogenesis is initiated by the side-chain cleavage of cholesterol catalyzed by CYP11A1 to yield the steroid precursor, pregnenolone. Pregnenolone passively diffuses to the endoplasmic reticulum where it is converted to progesterone by type II 3β-hydroxysteroid dehydrogenase (3βHSD2). Progesterone is then hydroxylated to 11-deoxycorticosterone by CYP17. The final late rate-limiting steps of aldosterone biosynthesis are completed in the mitochondria, where aldosterone synthase (CYP11B2) catalyzes the conversion of 11-deoxycorticosterone to corticosterone and subsequently to aldosterone.

Aldosterone biosynthesis occurs via a series of enzymatic reactions in the mitochondria and the endoplasmic reticulum of the zG cell and involves three cytochrome P450 enzymes and one hydroxysteroid dehydrogenase (Fig. 1). Cholesterol side-chain cleavage complex (CYP11A1) and aldosterone synthase (CYP11B2) are localized in the inner mitochondrial membrane, while 21-hydroxylase (CYP21) and type II 3β-hydroxysteroid dehydrogenase (3βHSD2) are found in the endoplasmic reticulum (8).

The primary precursor for aldosterone biosynthesis is cholesterol, which can be derived from several sources (9, 10): (1) de novo cholesterol synthesis, (2) circulating lipoprotein-derived cholesteryl esters via either “selective” uptake or receptor-mediated endocytosis, and (3) mobilization of stored cholesteryl esters via the actions of neutral cholesteryl ester hydrolase, also known as hormone-sensitive lipase. The first reaction in aldosterone biosynthesis is the mitochondrial conversion of cholesterol to pregnenolone. This step is tightly regulated by the steroidogenic acute regulatory (StAR) protein, which transports cholesterol from the outer to the inner mitochondrial membrane where CYP11A1 is located (11). Pregnenolone can then passively diffuse to the endoplasmic reticulum where it is converted to progesterone by 3βHSD2. Progesterone is then hydroxylated by CYP21 to 11-deoxycorticosterone. Finally, aldosterone biosynthesis is completed in the mitochondria, where deoxycorticosterone undergoes 11β- and 18-hydroxylation, followed by 18-oxidation. These final reactions are catalyzed by a single enzyme, aldosterone synthase, encoded by CYP11B2. Once synthesized, aldosterone is secreted from the zG cells. Thus, aldosterone biosynthesis involves two key rate-limiting steps: the early (acute) rate-limiting step requires the expression and phosphorylation of StAR protein (12, 13, 14), while the late (chronic) rate-limiting step involves the expression and regulation of CYP11B2 (15, 16).

To investigate the signaling mechanisms involved in aldosterone biosynthesis, several glomerulosa cell models are routinely used. These include primary cultures derived from different species (e.g., human, bovine, murine) and a few adrenocortical carcinoma cell lines (e.g., human H295R, an H295R clone, HAC15 (17), and Y1 mouse cells). (18, 19, 20, 21)

## Lipid second messengers

**Fig. 2:**
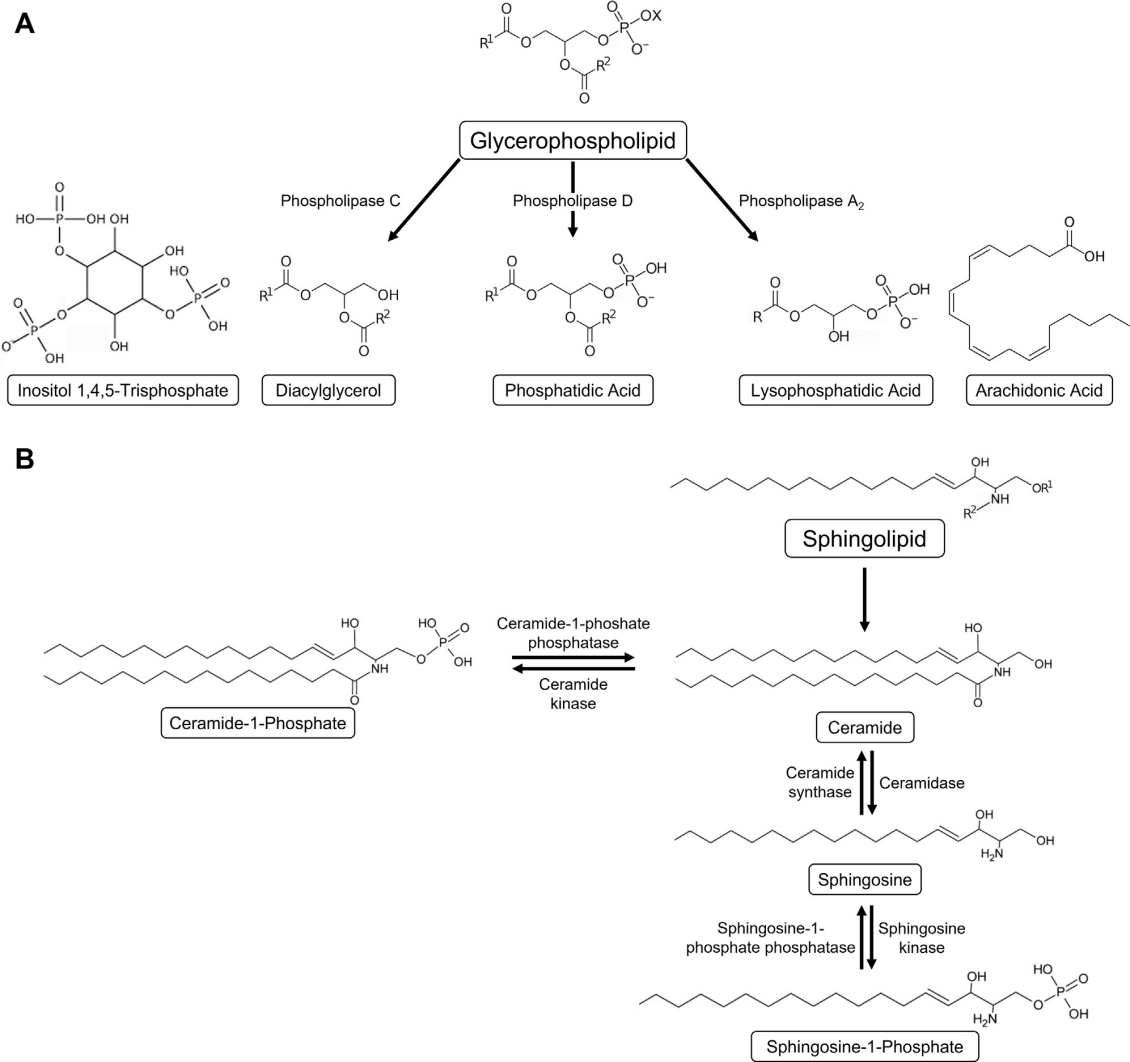
Lipid-derived second messengers. Hydrolysis of two classes of lipids, glycerophospholipids and sphingolipids, generates parallel series of lipid second messengers. A: Hydrolysis of glycerophospholipids yields diacylglycerol (and inositol 1,4,5-trisphosphate), phosphatidic acid, and lysophosphatidic acid (and free fatty acids such as arachidonic acid). B: Hydrolysis of sphingolipids results in the production of ceramide, ceramide-1-phosphate, sphingosine, and sphingosine-1-phosphate.

Phospholipids serve as integral structural components of cell membranes by spontaneously forming a lipid bilayer that maintains cell integrity. In addition, phospholipids serve as a reservoir of bioactive lipids involved in important signaling processes. Extracellular signals elicit the hydrolysis of two main classes of lipids to generate lipid second messengers: glycerophospholipids and sphingolipids. In the former class are included the phosphoinositides and phosphatidylcholine, with diacylglycerol (DAG) as the hydrophobic backbone, and the latter includes sphingomyelin, for which the hydrophobic backbone is ceramide. Signaling-induced hydrolysis of glycerophospholipids and sphingolipids generates parallel series of lipid second messengers (Fig. 2): lysophosphatidic acid (LPA), phosphatidic acid (PA), and DAG (and certain free fatty acids that can serve as cell signals themselves or as precursors to signaling molecules) versus sphingosylphosphorylcholine (SPC), ceramide-1-phosphate (C1P), ceramide, sphingosine, and sphingosine-1-phosphate (S1P). Lipid second messengers that retain two acyl chains, such as DAG, PA, C1P and ceramide, remain associated with the membrane while those that have only one acyl chain, such as LPA, SPC, S1P and sphingosine, are hydrophobic but can dissociate from membranes (22).

## Glycerophospholipid-derived second messengers

### Phosphoinositide signaling system: DAG and IP_3_

**Fig. 3:**
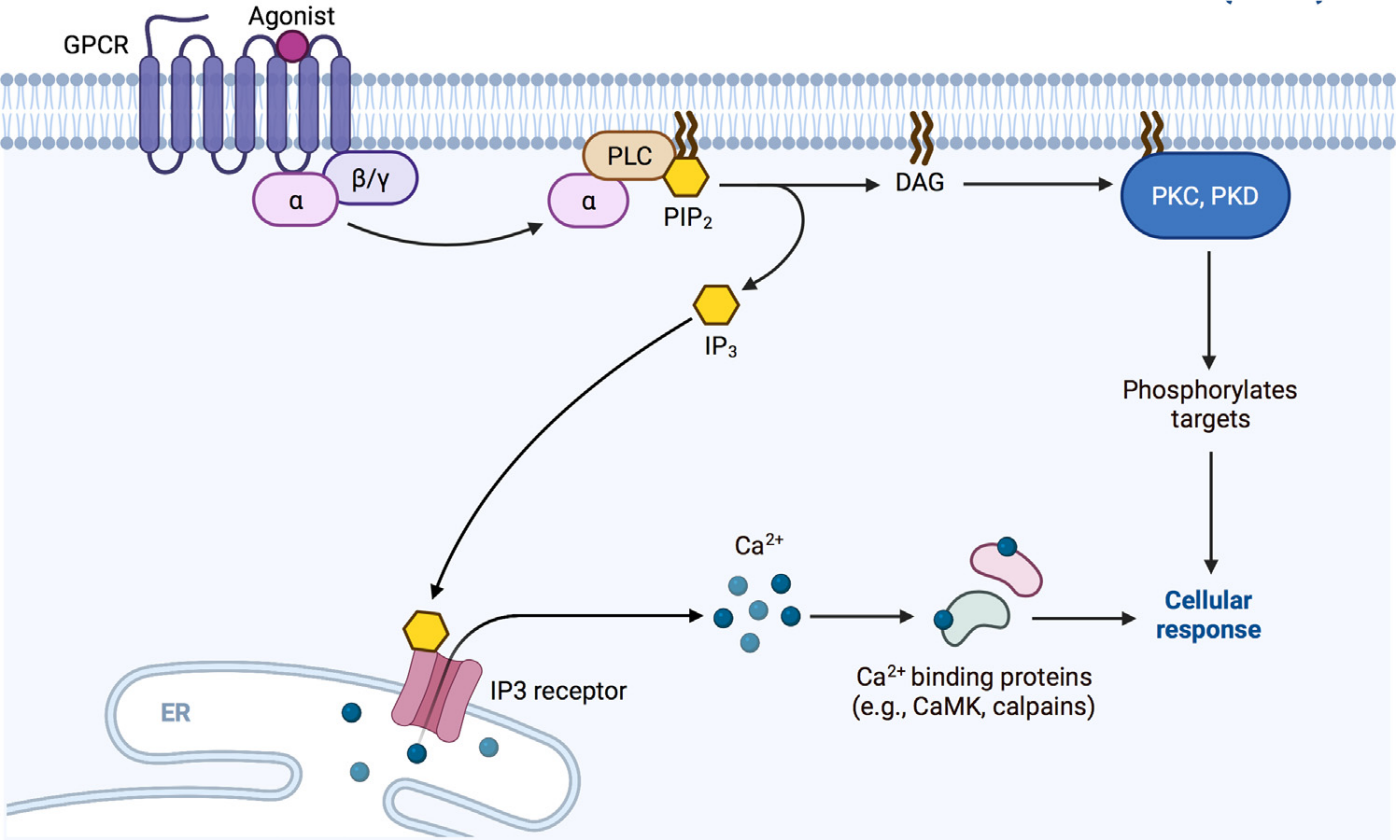
Phosphoinositide signaling system. Binding of agonists to G protein-coupled receptors (GPCRs) promotes the exchange of GTP for GDP on Gα subunits, which then bind and activate phospholipase C (PLC). PLC hydrolyzes phosphatidylinositol 4,5-bisphosphate (PIP_2_) to generate two second messengers, inositol 1,4,5-trisphosphate (IP_3_) and diacylglycerol (DAG). IP_3_ binds to IP_3_ receptors on the endoplasmic reticulum (ER) causing the release of calcium into the cytosol. The elevated cytosolic calcium levels can bind to and activate calcium/calmodulin-dependent protein kinase (CaMK), calpains, and classical (in conjunction with calcium) protein kinase C (PKC) isoenzymes. DAG remains in the membrane and activates proteins such as novel PKC isoenzymes and protein kinase D (PKD) isoenzymes. Figure adapted from “Activation of Protein Kinase C (PKC)”, created with BioRender.com (2021). Retrieved from https://app.biorender.com/biorender-templates.

The phosphoinositide signaling system, illustrated in Figure 3, is important in regulating steroidogenesis in adrenocortical cells in response to agonists. Ang II and elevated serum potassium levels are the primary physiological regulators of aldosterone production in the zG of the adrenal gland. Serum potassium signals through changes in zG membrane potential. Adrenal glomerulosa cells are usually hyperpolarized and maintain a negative resting membrane potential (−80 mV) close to the Nernst potential for potassium, which suggests that the membrane potential is determined mainly by the membrane potassium permeability. The potassium channels involved include the two pore-domain potassium channels, TWIK-related-acid-sensitive potassium family (TASK-1, TASK-3) and TWIK-related potassium channel 1, and the G protein-coupled, inwardly rectifying potassium channel Kir3.4 (23, 24, 25, 26, 27, 28). Elevated extracellular potassium levels depolarize the plasma membrane and activate the voltage-dependent T-type and L-type calcium channels (29, 30, 31, 32, 33, 34), leading to calcium influx and triggering signaling mechanisms described below for Ang II, including the activation of phospholipase D (PLD) (35).

The adrenal gland expresses two receptors for Ang II: Ang II receptor type 1 and Ang II receptor type 2; Ang II receptor type 1 is the primary receptor involved in Ang II-induced stimulation of aldosterone production (36). The primary event following ligand binding and receptor activation is the generation of DAG directly by phospholipase C (PLC). PLC cleaves the membrane phospholipid phosphatidylinositol 4,5-bisphosphate (PIP_2_) to yield DAG and inositol 1,4,5-trisphosphate (IP_3_). Interestingly, DAG can directly inhibit TASK-1 and TASK-3 channels causing a strong decrease of the fractional potassium conductance and depolarization of the membrane (37). Therefore, Ang II-induced depolarization, through inhibition of the two pore-domain potassium as well as G protein-coupled, inwardly rectifying potassium channels (23, 27, 33, 38, 39, 40), also activates voltage-gated T-type and L-type calcium channels resulting in calcium influx and increasing cytosolic calcium concentration (28, 29, 30, 31, 32, 41). Further, upon binding to its receptors expressed in the endoplasmic reticulum (42), IP_3_ is thought to initiate aldosterone production by inducing calcium release from the intracellular stores and eliciting a transient increase in cytosolic calcium concentration (43). This increase activates calcium/calmodulin-dependent protein kinases, which increase the transcription of steroidogenic enzymes involved in aldosterone biosynthesis (44, 45, 46). DAG remains in the membrane to activate DAG-responsive enzymes, such as protein kinase C (PKC) and protein kinase D (PKD) (47, 48, 49). Signals transduced through PIP_2_ hydrolysis are terminated by the metabolism of IP_3_ and DAG into derivatives lacking second messenger capabilities. DAG can also be phosphorylated to PA, which itself has signaling properties or can be deacylated by an arachidonate-preferring DAG lipase, releasing arachidonic acid (AA) for conversion to eicosanoids, such as the aldosterone-promoting 12-HETE (50, 51, 52, 53, 54) (refer to section LPA and AA). Inositol tetrakisphosphate formed by phosphorylation of IP_3_ may act as a second messenger to further regulate calcium homeostasis (55).

Larson *et al.* first demonstrated the incorporation of radiolabeled phosphate into phosphatidylinositol and polyphosphoinositides in the zG by treating rat adrenal capsules in vitro with Ang II and elevated potassium levels (56). Subsequent studies by Rasmussen *et al.* showed that Ang II induced a rapid hydrolysis of phosphatidylinositol 4-phosphate and PIP_2_ resulting in sustained production of inositol bisphosphate, IP_3_, and DAG in adrenal glomerulosa cells (43). Ang II was also shown to induce an increase in absolute DAG levels in freshly isolated and cultured bovine adrenal glomerulosa cells (57, 58, 59) This Ang II-induced DAG increase is biphasic, with an initial peak followed by a transient decrease; the second increase is higher in magnitude than the first increase (57). Moreover, the DAG produced by Ang II stimulation consists of multiple species, for example, those containing the fatty acids arachidonate or myristate. Following the increase in DAG formation upon Ang II binding, the subsequent addition of an Ang II antagonist results in a rapid decline in arachidonate-DAG, but a slower decline in myristate-DAG, suggesting that the species of DAG generated might play a role in the speed with which signaling is terminated (58).

In addition to conventional aldosterone secretagogues, VLDL, a nonconventional aldosterone secretagogue (60), induces an increase in radiolabeled DAG levels, and a PLC inhibitor, U-73122, blocks this increase in HAC15 cells. In addition, an IP_3_ receptor inhibitor decreases VLDL-induced aldosterone production in HAC15 cells (48). U-71322 also inhibits the parathyroid hormone (PTH)- and PTH-related peptide-induced elevation in radiolabeled IP_3_ release and aldosterone secretion from dispersed human adrenocortical cells (61). PTH and PTH-related peptide, members of the vasoactive intestinal peptide-secretin-glucagon family of peptides, are well-known paracrine modulators of the secretory activity of the adrenal cortex (62, 63). Yet, another nonconventional aldosterone secretagogue is S1P (64). Sewer *et al.* showed that S1P-induced StAR transcription requires PLC activation and results in the accumulation of cytoplasmic IP_3_ in H295R cells (65).

Together, these data suggest the importance of phosphoinositide turnover to aldosterone synthesis.

### DAG and PA

In addition to being directly produced by PLC-mediated cleavage of phospholipids, DAG can be generated indirectly by the action of PLD. PLD is an enzyme that hydrolyzes phosphatidylcholine to produce PA (phosphorylated DAG), also a lipid second messenger. PA can then be dephosphorylated by lipid phosphate phosphatases (LPPs; also known as phosphatidate phosphatases or the lipin family) to yield DAG (66). The phospholipid source of the DAG dictates the species of DAG generated. Since phosphatidylcholine is enriched in myristate, Ang II-induced increases in myristate-DAG in adrenal glomerulosa cells support the role of PLD in Ang II-elicited DAG generation (58). In turn, numerous reports demonstrate the importance of PLD activity in the aldosterone secretory response to Ang II and other secretagogues (64, 66, 67, 68, 69).

PA has been proposed to function as a slow-release reservoir of DAG for sustained aldosterone production (66, 70, 71). Bollag *et al.* demonstrated that Ang II elicits increases in the levels of radiolabeled PA and phosphatidylethanol (PEt; in the presence of ethanol) in cultured bovine adrenal glomerulosa cells (66). Phosphatidylcholine undergoes transphosphatidylation in the presence of ethanol to yield PEt, rather than hydrolysis to generate PA. PEt is not readily metabolized to DAG. The ability of ethanol to divert product formation away from PA (and DAG) in response to hormonal stimulation indicates that hormone-elevated DAG, in part, originates from the combined activity of PLD and LPP. Other primary alcohols can be used as well; PLD can utilize 1-butanol in place of water to form phosphatidylbutanol instead of PA. Phosphatidylbutanol also is not readily metabolized to DAG. Therefore, 1-butanol can be used to inhibit PLD-mediated lipid signal generation. Bollag *et al.* showed that 1-butanol inhibited Ang II-induced increases in PA and DAG levels as well as aldosterone secretion in bovine adrenal glomerulosa cells (67). S1P-stimulated aldosterone secretion in zG cells is also inhibited by primary alcohols (64). Combined PLD/LPP activity underlying sustained DAG production has also been observed using propranolol in terms of both S1P-induced aldosterone production in bovine adrenal glomerulosa cells (64) and Ang II-induced cortisol production in zona fasciculata cells of bovine adrenal glands (72). Propranolol, in addition to being a β-blocker, inhibits LPP (73, 74).

**Fig. 4:**
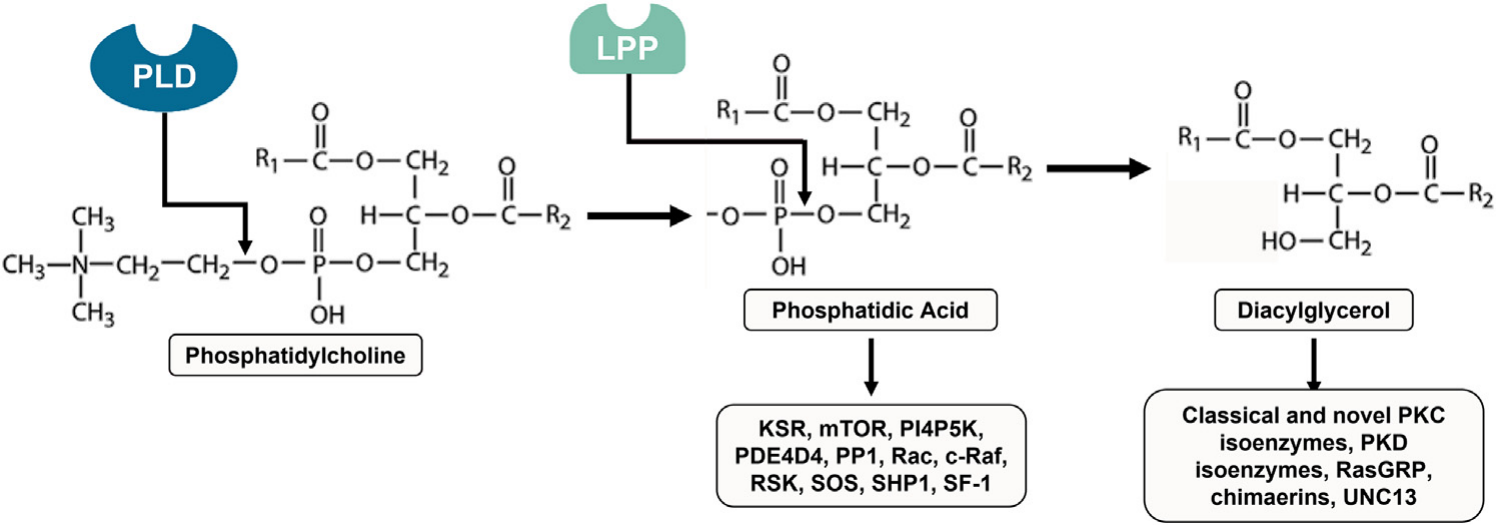
Downstream effectors of phospholipase D-generated lipid second messengers. Phospholipase D (PLD) hydrolyzes phosphatidylcholine to produce choline and phosphatidic acid (PA). PA can interact and modulate the activity of various downstream effectors such as the kinase suppressor of Ras (KSR), mammalian target of rapamycin (mTOR), phosphatidylinositol 4-phosphate 5-kinase (PI4P5K), phosphodiesterase 4D3 (PDE4D3), protein serine/threonine phosphatase 1 (PP1), the small GTP-binding proteins Rac and c-Raf, ribosomal S6 kinase (RSK), son of sevenless (SOS), Src homology region 2 domain-containing phosphatase-1 (SHP1), and nuclear hormone receptor steroidogenic factor-1 (SF-1). PA can be dephosphorylated by lipid phosphate phosphatases (LPPs) to yield diacylglycerol (DAG). DAG effectors include the classical and novel protein kinase C (PKC) isoenzymes, protein kinase D (PKD) family of protein kinases, the Ras guanine nucleotide release proteins (RasGRP), the Rac GTPase-activating proteins chimaerins, and UNC13 proteins.

The involvement of a particular PLD-generated lipid signal, PA or DAG, in mediating aldosterone production is not entirely clear (Fig. 4). PLD-generated DAG clearly contributes to Ang II- and S1P-induced aldosterone production in zG cell models (64, 66, 67, 71). DAG can, in turn, activate PKC isoforms, the PKD family of protein kinases, and Ras guanine nucleotide-releasing protein 1-3 (Ras guanine nucleotide exchange factors). DAG is an allosteric activator of PKC (75). In bovine adrenal glomerulosa cells, a PKC-activating phorbol ester, phorbol 12-myristate 13-acetate or PMA, is able to activate PLD while selective PKC inhibitors partially block PLD activation by Ang II and S1P. Moreover, PMA does not enhance PLD activation by Ang II or S1P; this lack of an additive effect with PMA suggests that both Ang II and S1P function to stimulate PLD activity through PKC. In addition, these results suggest that not only is PKC sufficient to activate PLD but also that it is necessary for agonist-induced PLD activation (64, 67). The PLC/DAG/PKC pathway can also activate PKD (as shown in Swiss 3T3 cells) (76), and Ang II-mediated PKD phosphorylation/activation is mediated, in part, by PLD activity (68), as well as by PKC and Src family kinases (49, 77) in primary bovine adrenal glomerulosa and H295R cells. PKD in turn phosphorylates and activates activating transcription factor (ATF)/cAMP response element binding (CREB) protein transcription factors to induce the transcription of StAR and aldosterone synthase and mediate acute Ang II-aldosterone secretion, as demonstrated in H295R cells and primary bovine adrenal glomerulosa cells (47, 77, 78).

However, PA is itself a lipid second messenger with downstream effectors, such as mammalian target of rapamycin, phosphodiesterase, phosphoinositide-synthesizing enzymes, protein phosphatase 1 (PP1), ribosomal S6 kinase (RSK), and steroidogenic factor-1 (SF-1). Elevated potassium levels and Ang II have been shown to dose-dependently increase the concentrations of PA in rat adrenal capsules in vitro (56). In addition to being generated by PLD, PA can also be produced by DAG kinase θ (DGKθ), which phosphorylates DAG formed by phosphoinositide turnover. ACTH/cAMP signaling stimulates DGKθ-catalyzed nuclear PA production in H295R cells (79, 80). Silencing DGKθ represses both basal and cAMP-dependent expression of genes (and proteins) involved in cholesterol mobilization (StAR, scavenger receptor type B class 1, low density lipoprotein receptor, hormone-sensitive lipase) and steroidogenesis (CYP11A1, 3βHSD2). In contrast, CYP21 gene and protein expression are increased in these DGKθ knockdown cells (81). PA acts as an endogenous ligand for SF-1 (79), a nuclear hormone receptor important in steroidogenesis (82). In line with the DGKθ knockdown results, PA has been shown to activate multiple SF-1 steroidogenic targets including CYP11A1, 3βHSD, CYP21, and CYP11B1/2 (79). SF-1 is also known to positively regulate StAR gene expression (83, 84), whereas it appears to negatively regulate CYP21 and CYP11B2 expression as well as aldosterone production (82, 85). However, a threshold level of SF-1 is required for basal CYP11B2 expression, with an elevation of SF-1 above this baseline leading to repression of CYP11B2 expression (82).

PA is also a potent and selective inhibitor of PP1 (86). PP1 is expressed in whole adrenals as well as the capsule, and its inhibition by nonselective inhibitors significantly reduces ACTH-stimulated aldosterone production in zG cells (87). Finally, in patients with primary aldosteronism (idiopathic hyperaldosteronism and aldosterone-producing adenomas), enhanced phosphorylation of mammalian target of rapamycin and RSK, downstream effectors of PA, correlates with plasma aldosterone levels (88). Therefore, whether PA or DAG or both of these lipid second messengers are important for aldosterone production remains to be determined.

### LPA and AA

PA can also be deacylated by phospholipase A_2_ (PLA_2_) to yield LPA and a free fatty acid such as AA. Bovine adrenal glomerulosa cells express G protein-coupled receptors for LPA that are predominantly coupled to G_i_ (89). LPA has been shown to have a mitogenic effect in bovine adrenal glomerulosa cells (similar to Ang II), which is completely prevented by pertussis toxin (PTX) (90). PTX ADP ribosylates and inhibits G_i_ in the bovine adrenal glomerulosa cell preparation (91, 92). Shah *et al.* showed that LPA causes proliferation of these cells through activation of Src and phosphoinositide-3-kinase (89) A selective Src family inhibitor, PP2, has been shown to inhibit Ang II-, potassium-, and dibutyryl-cAMP-stimulated aldosterone production through the induction of CYP17 in H295R cells (49, 93). LPA can also stimulate aldosterone secretion (64), and this ability is partially dependent upon epidermal growth factor receptor transactivation (89). LPA induces the activation of ERK1/2 and its downstream protein, RSK-1, and this activation is blocked by PTX (89). Further, PKC depletion by PMA-mediated downregulation in rat adrenal glomerulosa cells (94) partly attenuates LPA responses and a pan-PKC inhibitor, Gö6983, reduces LPA-induced ERK1/2 activation. These effects indicate that LPA exerts its action through two distinct signaling cascades: the epidermal growth factor receptor and PKC (89). LPA has also been shown to inhibit the TASK-1 channel, a two pore-domain potassium channel, in oocytes (39); Barrett *et al.* have demonstrated that zG-specific deletion of this TASK-1 channel in mice can produce mild autonomous hyperaldosteronism that is independent of the renin-angiotensin system and can result in chronic blood pressure elevation (25).

The other product of PLA_2_ activity, free fatty acids like AA, can also function as second messengers. AA can also be released upon DAG hydrolysis by DAG lipase (51, 53). AA specifically inhibits Ang II type 1 receptors but exerts no effect on Ang II type 2 receptors in bovine adrenal glomerulosa cells (95). Exogenous AA stimulates both radiocalcium efflux and aldosterone secretion in bovine adrenal glomerulosa cells (96). A trifluoromethylketone analog of AA and inhibitor of cytosolic PLA_2_, AACOCF_3_, raises basal aldosterone secretion in dispersed rat zG and human adrenocortical cells (97). AA can be subsequently metabolized by the lipoxygenase and the cyclooxygenase pathways in zG cells (98). It seems that the lipoxygenase, but not the cyclooxygenase, products of AA metabolism, play a role in aldosterone secretion as positive feed forward mediators (52, 96, 99, 100, 101, 102). Isolated rat adrenal glomerulosa cells and normal human adrenal glomerulosa cells can produce the lipoxygenase products, 12- and 15-HETE, in the basal state. Ang II, but not ACTH or potassium, selectively stimulates 12-HETE production. Selective and nonselective lipoxygenase inhibitors block Ang II-mediated intracellular calcium increases and aldosterone secretion as well as the formation of 12-HETE, and these responses can be restored by the addition of exogenous 12-HETE in isolated rat adrenal glomerulosa cells and cultured aldosterone-producing adenoma cells (50, 52, 54). 12-HETE increases aldosterone production, in part, through activation of CREB/ATF-1 and the p38 mitogen-activated protein kinase pathway in H295R cells (103). Ang II-mediated aldosterone secretion is unaltered by 5-lipoxygenase inhibitors (50). Nadler *et al.* showed a similar effect in aldosterone-producing adenomas (52). Podestá *et al.* highlighted the role of the generation and export of intramitochondrial AA in regulating the induction of StAR protein in H295R cells (104). In this mechanism, acyl-CoA synthetase long chain family member 4 (ACSL4) esterified free AA to yield AA-CoA, which can be delivered to mitochondrial acyl-CoA thioesterase 2 to release AA into the mitochondria upon hormone treatment. Ang II and potassium regulate the expression of ACSL4 and acyl-CoA thioesterase 2, with the induction of ACSL4 dependent on protein tyrosine phosphatases (PTPs). The site of action of PTPs precedes activation of cholesterol transport into the mitochondria. Therefore, aldosterone synthesis by Ang II can be linked to the sequential actions of PTP, ACSL4, and StAR protein. However, additional studies are needed to elucidate the role of LPA and AA metabolites in the regulatory mechanisms of aldosterone synthesis.

## Sphingolipid-derived second messengers

Sphingolipid metabolism and steroidogenesis have a reciprocal relationship in many respects. In H295R cells, both ACTH and dibutyryl-cAMP stimulate sphingolipid metabolism by rapidly promoting the catabolism of sphingomyelin, ceramide, and sphingosine, with a concomitant increase in S1P secretion (105).

Brizuela *et al.* showed in bovine adrenal glomerulosa cells that short-chain cell permeable ceramides such as C_6_-ceramide, which are potent inhibitors of PLD (106, 107), not only blocked PLD stimulation by S1P but also completely abolished S1P-induced aldosterone secretion (64). Ceramide has no effect on cAMP production but has been shown to abrogate StAR mRNA and protein expression in 8Br-cAMP-treated MA-10 Leydig cells and in human chorionic gonadotropin-stimulated adult Sprague Dawley rats (108, 109, 110). However, the precise molecular mechanism(s) by which ceramide regulates aldosterone secretion is unclear.

Ceramide can be phosphorylated to C1P by ceramide kinase. C1P has been reported to be a specific and potent inducer of AA release and since AA plays a role in steroidogenesis (refer to LPA and AA), C1P may be a regulator of steroidogenesis (111). However, this sphingolipid has been little studied in the zG.

Ceramide can also be hydrolyzed to sphingosine by ceramidases. Sphingosine serves as an antagonist of SF-1 to modulate steroidogenic gene transcription. Under basal conditions, sphingosine is bound to SF-1, and this binding antagonizes cAMP activation of CYP17 gene transcription in H295R cells (112). PA has been demonstrated to be an endogenous activating ligand for SF-1 (79), with cAMP promoting the displacement of sphingosine (and SPC; see below) from and the binding of PA to the SF-1 ligand-binding pocket (112). The sphingosine-generating enzyme, acid ceramidase (ASAH1), directly regulates the intracellular balance of ceramide, sphingosine, and S1P. In H295R cells, suppression of ASAH1 can induce the expression of steroidogenic genes CYP17A1, CYP11A1, CYP21A2, CYP11B1, CYP11B2, StAR, and hormone-sensitive lipase (113). Silencing ASAH1 mimics cAMP-stimulated CYP17A1 transcription, supporting ASAH1’s role in regulating the function of SF-1 and therefore, steroidogenic gene expression. SPC (i.e., lysosphingomyelin) can also bind to SF-1 but with a lesser affinity than sphingosine under basal conditions, and cAMP treatment also promotes its dissociation from the receptor. However, SPC has no effect on the ability of cAMP or SF-1 to promote CYP17 expression (112).

Sphingosine kinases can phosphorylate sphingosine to form S1P. S1P can exert its signaling functions both extracellularly and within the cell (114, 115). Extracellularly, S1P binds to various G protein-coupled S1P receptors (S1PR_1-5_) through which it serves as a stimulator of aldosterone secretion and PLD activity in bovine adrenal glomerulosa cells (64). Protein kinase B (also known as Akt) and ERK1/2 have been identified as S1P targets. Brizuela *et al.* proposed a working model in bovine adrenal glomerulosa cells wherein S1P stimulates phosphoinositide-3-kinase/protein kinase B and mitogen-activated kinase kinase/ERK pathways, leading to PLD activation. S1P also causes an influx of calcium and activation of PKCα and PKCδ, which are upstream of PLD (116).

ACTH and cAMP can stimulate an increase in intracellular S1P in H295R cells (105). S1P, in turn, can increase CYP17 mRNA expression by promoting the cleavage and nuclear localization of sterol regulatory element binding protein 1 (SREBP1) (117). Intracellular S1P pools are mainly regulated by S1P lyase 1 (SGPL1); SGPL1 executes the final decisive step in sphingolipid catabolism by initiating the irreversible breakdown of S1P into ethanolamine phosphate and hexadecenal. Loss-of-function mutations in SGPL1 have been associated with primary adrenal insufficiency and steroid-resistant nephrotic syndrome in humans (118, 119, 120). SGPL1 is expressed in normal human adrenals while adrenals from *Sgpl*^*-/-*^ mice shows compromised cortical zonation with less definition between the zG and zona fasciculata. *Sgpl*^*-/-*^ mouse adrenals also show lower expression of CYP11A1, while the characteristic patchy expression of aldosterone synthase (CYP11B2) in wild-type mice is replaced by a more continuous pattern of expression (119).

These studies highlight the importance of sphingolipid metabolites in aldosterone synthesis and secretion.

## Conclusion

In conclusion, cumulative evidence points toward an important role of lipid second messengers in the regulation of aldosterone production. These bioactive lipids serve as potential links between the agonists binding to their receptors and the synthesis and secretion of aldosterone. The studies discussed in this review also highlight the need for further investigation to elucidate the role of DAG, PA, LPA, and sphingolipid metabolites in aldosterone production.

## Conflict of interest

No author has an actual or perceived conflict of interest with the content of this article.
